# Supplementary material for: Effect of osteoporosis-related reduction in the mechanical properties of bone on the acetabular fracture during a sideways fall: A parametric finite element approach
Source: PLoS One. 2022 Feb 7;17(2):e0263458. doi: 10.1371/journal.pone.0263458 (PMC8820641; doi:10.1371/journal.pone.0263458)
Supplement: S1 File — (DOCX) [file pone.0263458.s001.docx]

Effect of osteoporosis-related reduction in the mechanical properties of bone on the acetabular fracture during a sideways fall: A parametric finite element approach

Shahab Khakpour ^1¶,^*, Amir Esrafilian ^2¶^, Petri Tanska ^2&^, Mika E. Mononen ^2&^, Rami K. Korhonen ^2&^, and Timo Jämsä ^1,3,4¶^

1. Research Unit of Medical Imaging, Physics, and Technology, University of Oulu, Oulu, Finland.
2. Department of Applied Physics, University of Eastern Finland, Kuopio, Finland.
3. Medical Research Center, University of Oulu and Oulu University Hospital, Oulu, Finland.
4. Diagnostic Radiology, Oulu University Hospital, Oulu, Finland.

***Appendix A. Mechanical Properties of Tissues and Ground***

Table A1. Empirical relationships between mechanical properties of cortical and trabecular bone with the apparent density and strain rate [1].

| **Cortical** | Compressive (c) | Tensile (t) | **Trabecular** | Compressive (c) | Tensile (t) |
| --- | --- | --- | --- | --- | --- |
| Proportionality Stress limit ($\sigma_{p,cort})$ | ${0.8 \times\sigma}_{y,c}$ | ${0.8 \times\sigma}_{y,t}$ | Proportionality Stress limit ($\sigma_{p,trab})$ | ${0.8 \times\sigma}_{y,c}$ | ${0.8 \times\sigma}_{y,t}$ |
| Proportionality Strain limit ($\varepsilon_{p,cort})$ | $\sigma_{p,c}/E$ | $\sigma_{p,t}/E$ | Proportionality Strain limit ($\varepsilon_{p,trab})$ | $\sigma_{p,c}/E$ | $\sigma_{p,t}/E$ |
| Yield stress ($\sigma_{y,cort})$ | $137.5 \times\alpha_{y,c}(\dot{\varepsilon)\times} \frac{49.5\times\rho_{app}^{2}}{181.9}$ | $103.7 \times\alpha_{y,t}(\dot{\varepsilon)\times} \frac{49.5\times\rho_{app}^{2}}{124.9}$ | Yield stress ($\sigma_{y,trab})$ | $\sigma_{u,c,trab}/1.1$ | $\sigma_{u,t,trab}/1.1$ |
| Yield Strain ($\varepsilon_{y,cort})$ | $0.0012\times\beta_{y,c}(\dot{\varepsilon)}$ | $0.0086\times\beta_{y,t}(\dot{\varepsilon)}$ | Yield Strain ($\varepsilon_{y,trab})$ | $\sigma_{y,c,trab}$/E+0.002 | $\varepsilon_{y,trab}\times0.7$ |
| Ultimate Stress ($\sigma_{u,cort})$ | $49.5\times\rho_{app}^{2}\times\alpha_{u,c}(\dot{\varepsilon)}$ | $49.5\times\rho_{app}^{2}\times\alpha_{u,c}(\dot{\varepsilon)}\times0.68$ | Ultimate Stress ($\sigma_{u,trab})$ | $49.5\times SRS\times\rho_{app}^{2}$ | $49.5\times SRS\times\rho_{app}^{2}\times0.7$ |
| Ultimate Strain ($\varepsilon_{u,cort})$ | $0.0164\times\beta_{u,c}(\dot{\varepsilon)}$ | $0.0276\times\beta_{u,t}(\dot{\varepsilon)}$ | Ultimate Strain ($\varepsilon_{u,trab})$ | 0.2 | 0.014 |

**Strain rate scaling formulas proposed by Enns-Bray et al.** [1]

$SRS={(\frac{\dot{\varepsilon}}{0.005})}^{0.06}$

Cortical stiffness

$$E_{C}^{Co}=1+.0.0163\times min (\dot{\varepsilon},30)$$

$$E_{T}^{Co}=1+.0.0297\times min (\dot{\varepsilon},18)$$

Tensile properties

$$\alpha_{u,t} \left( \dot{\varepsilon} \right)=-2.093\times{10}^{-5}\times{\min\left( \dot{\varepsilon},18 \right)}^{3}+1.420\times{10}^{-3}\times{\min\left( \dot{\varepsilon},18 \right)}^{2}-5.082\times{10}^{-2}\times min \left( \dot{\varepsilon},18 \right)+1$$

$$\alpha_{y,t} \left( \dot{\varepsilon} \right)=-1.424\times{10}^{-5}\times{\min\left( \dot{\varepsilon},18 \right)}^{3}+4.395\times{10}^{-4}\times{\min\left( \dot{\varepsilon},18 \right)}^{2}-3.466\times{10}^{-2}\times min \left( \dot{\varepsilon},18 \right)+1$$

$\beta_{u,t} \left( \dot{\varepsilon} \right)=-3.613\times{10}^{-6}\times{\min\left( \dot{\varepsilon},18 \right)}^{5}+2.034\times{10}^{-4}\times{\min\left( \dot{\varepsilon},18 \right)}^{4}-4.501\times{10}^{-3}\times{\min\left( \dot{\varepsilon},18 \right)}^{3}+4.967\times{10}^{-2}\times{\min\left( \dot{\varepsilon},18 \right)}^{2}-0.280\times min \left( \dot{\varepsilon},18 \right)+1$

$\beta_{y,t} \left( \dot{\varepsilon} \right)=9.005\times{10}^{-6}\times{\min\left( \dot{\varepsilon},18 \right)}^{4}-2.819\times{10}^{-4}\times{\min\left( \dot{\varepsilon},18 \right)}^{3}+2.626\times{10}^{-3}\times{\min\left( \dot{\varepsilon},18 \right)}^{2}+-0.039\times min \left( \dot{\varepsilon},18 \right)+1$

Compressive properties

$$\alpha_{u,c} \left( \dot{\varepsilon} \right)=-1.028\times{10}^{-3}\times{\min\left( \dot{\varepsilon},30 \right)}^{2}+3.063\times{10}^{-2}\times min \left( \dot{\varepsilon},30 \right)+1$$

$$\alpha_{y,c} \left( \dot{\varepsilon} \right)=-1.920\times{10}^{-2}\times min \left( \dot{\varepsilon},30 \right)+1$$

$$\beta_{u,c} \left( \dot{\varepsilon} \right)=-8.830\times{10}^{-3}\times{\min\left( \dot{\varepsilon},30 \right)}^{2}+0.303\times min \left( \dot{\varepsilon},30 \right)+1$$

$\beta_{y,c} \left( \dot{\varepsilon} \right)=-2.463\times{10}^{-2}\times min \left( \dot{\varepsilon},30 \right)+1$

| Cortical Bone (MAT_FU_CHANG_FOAM (083)) | | | | |
| --- | --- | --- | --- | --- |
| *Density (kg/m^3)* | ***Young's modulus (MPa)*** | ***Rayleigh damping coefficient*** | ***Table ID for nominal stress-strain data as a function of strain rate.*** | |
| 1,800 | 1.648E4 | 0.1 | 12 (Fig. A1) | |
| *Strain rate evaluation flag* | | *Tensile stress evaluation* | |  |
| Principal strain rates for each principal direction | | input via load curves with the tensile response corresponds to negative values of stress and strain | |  |

**
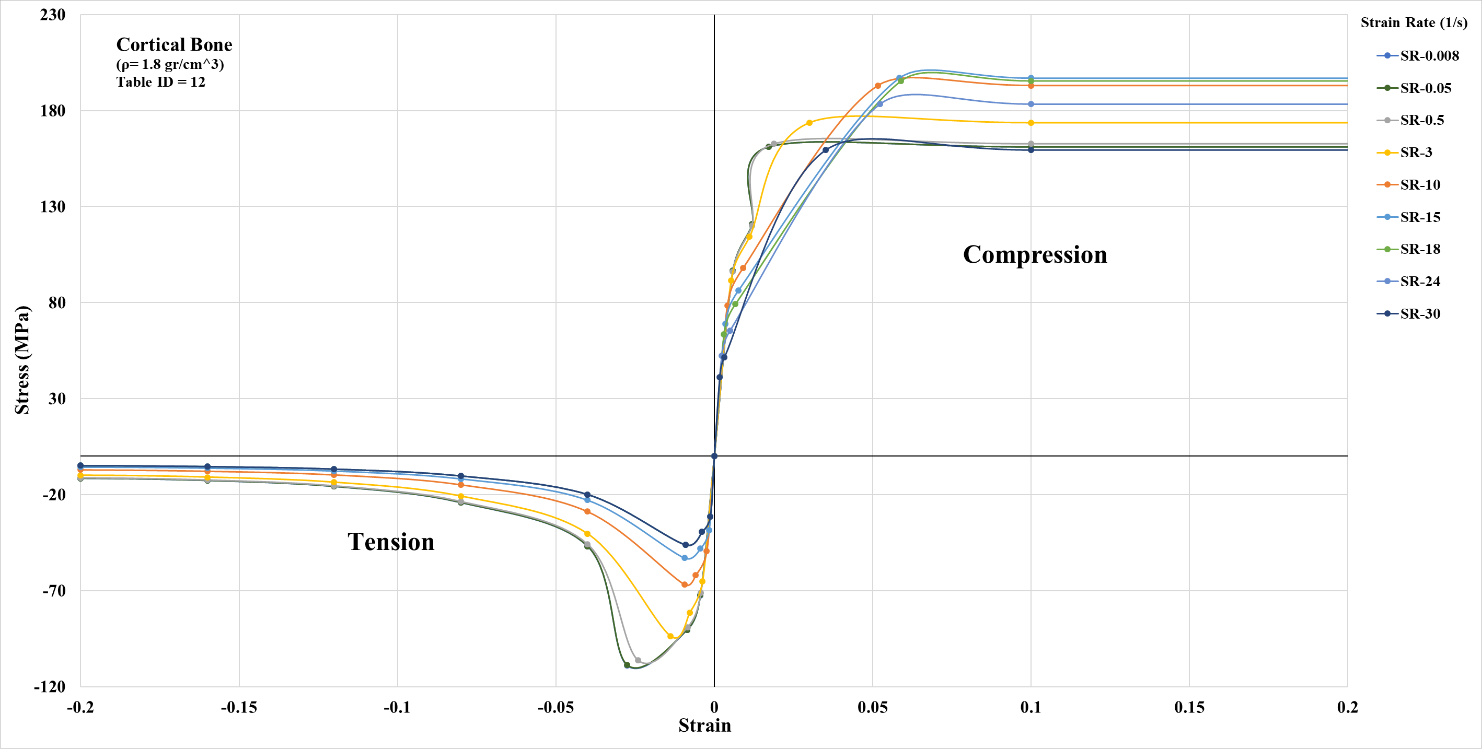

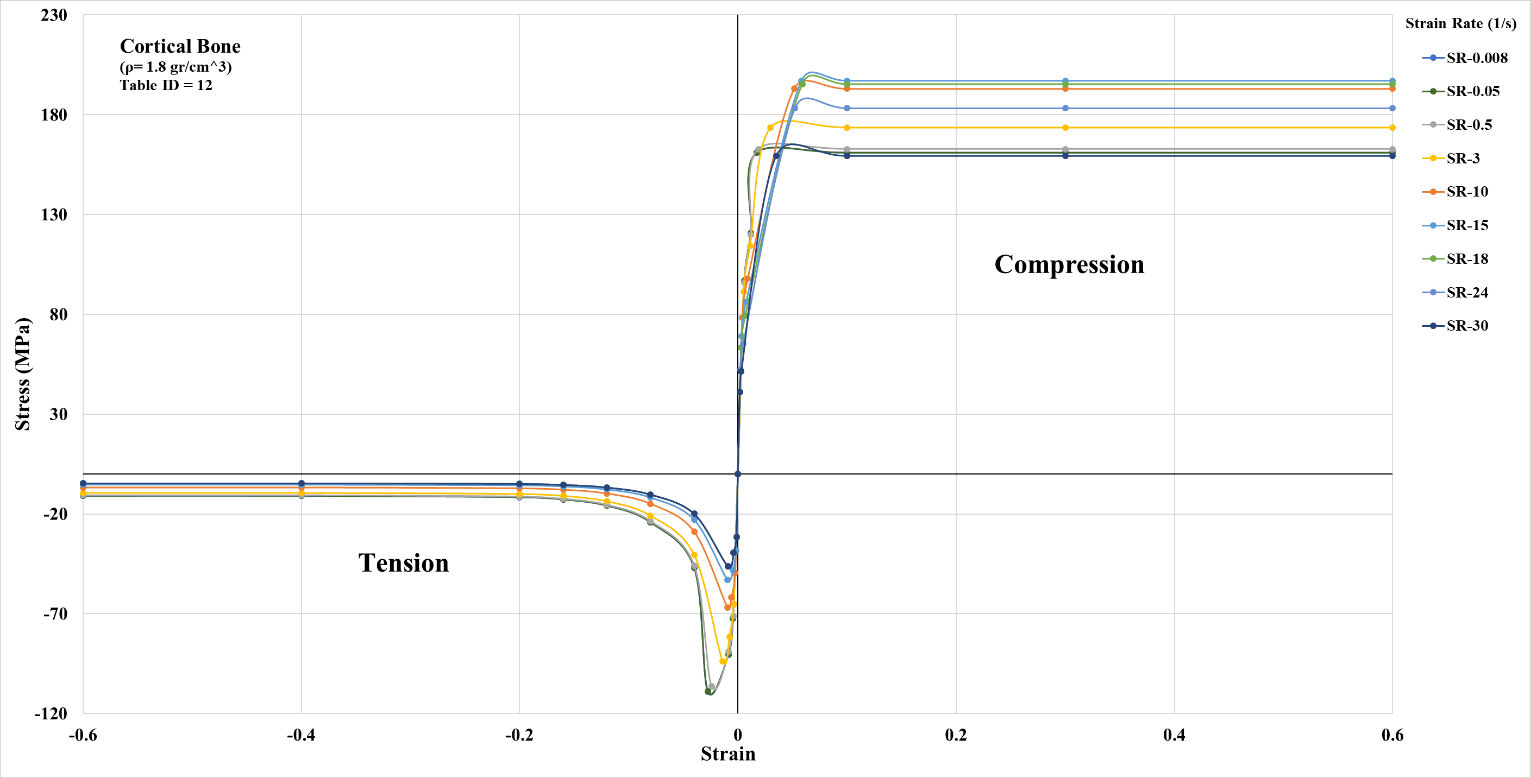
**

***
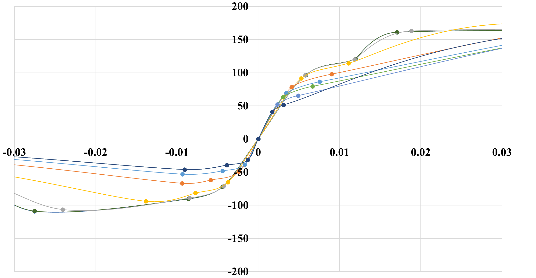
***

Figure A1. Nominal stress-strain curves for cortical bone (Table ID=12)

| Trabecular Bone-T5 (ρ=4.7E-10) (MAT_FU_CHANG_FOAM (083)) | | | | |
| --- | --- | --- | --- | --- |
| *Density (kg/m^3)* | ***Young's modulus (MPa)*** | ***Rayleigh damping coefficient*** | ***Table ID for nominal stress-strain data as a function of strain rate.*** | |
| 470 | 2.223E3 | 0 | 405 (Fig. A2) | |
| *Strain rate evaluation flag* | | ***Tensile stress evaluation*** | |  |
| Principal strain rates for each principal direction | | input via load curves with the tensile response corresponds to negative values of stress and strain | |  |


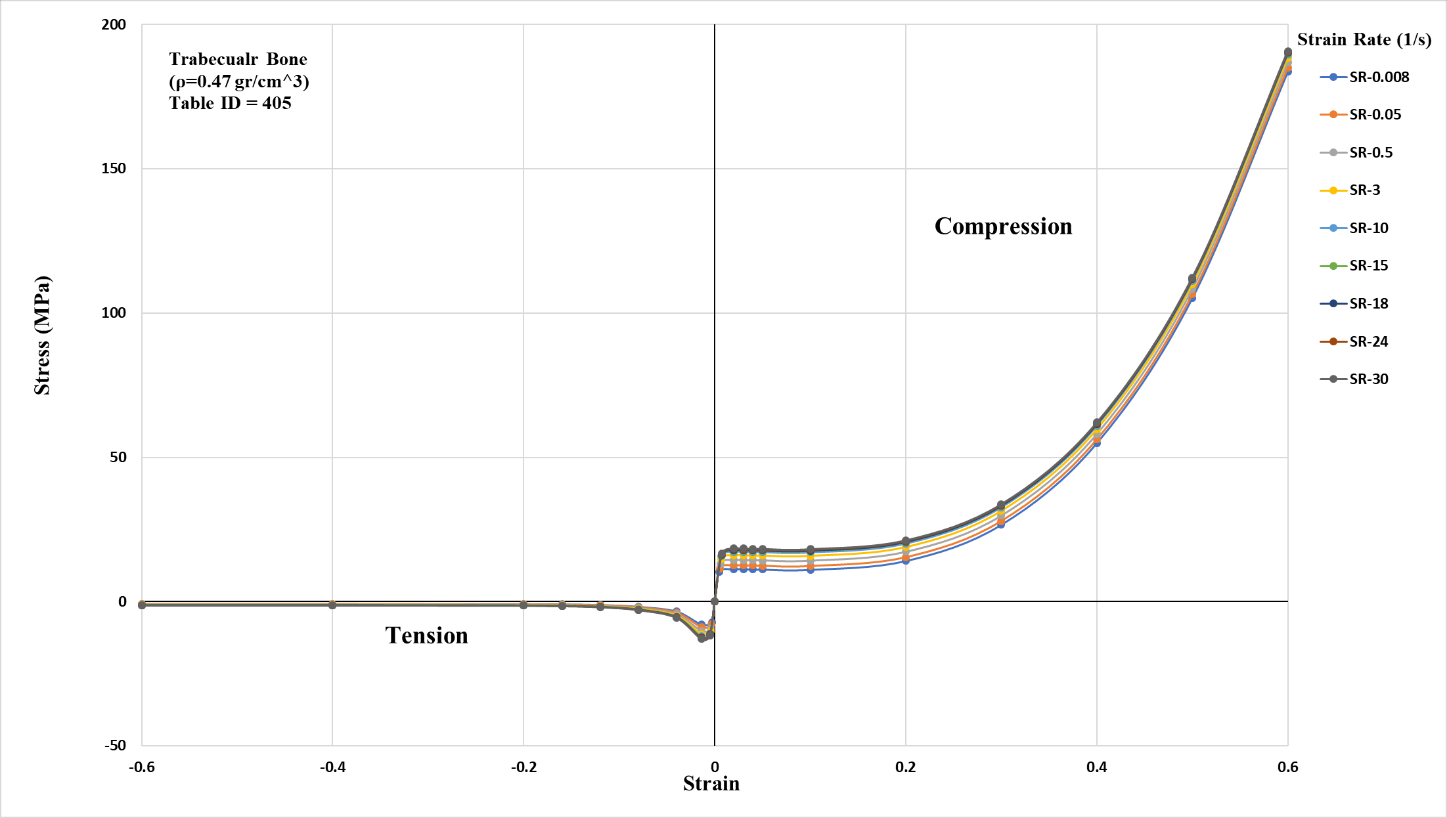

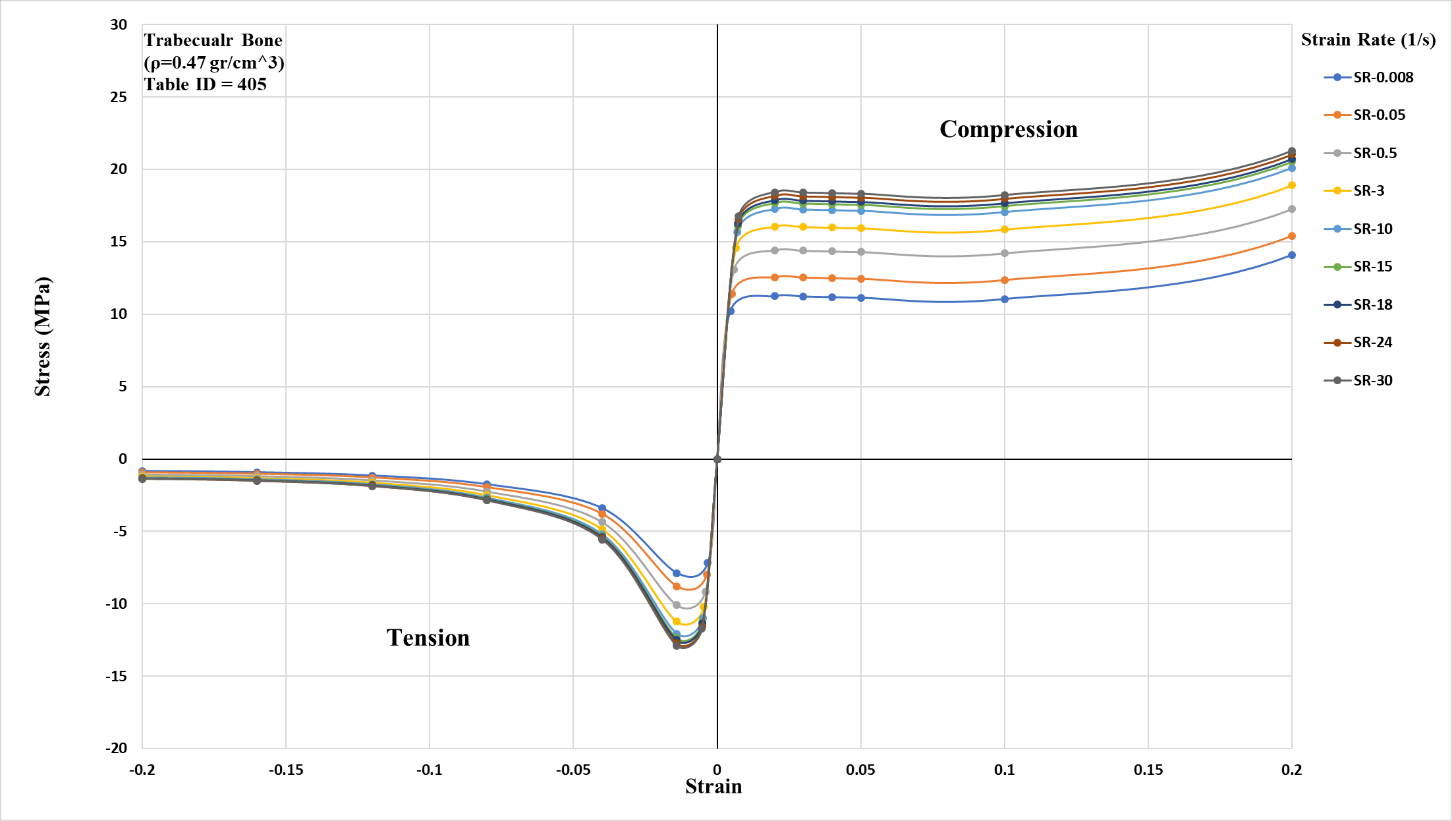

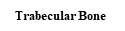

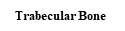


Figure A2. Nominal stress-strain curves for trabecular bone- Density =0.47 g/cm^3^ (Table ID=405)

| Trabecular Bone-T9 (ρ=8.5E-10) (MAT_FU_CHANG_FOAM (083)) | | | | |
| --- | --- | --- | --- | --- |
| *Density (kg/m^3)* | ***Young's modulus (MPa)*** | ***Rayleigh damping coefficient*** | ***Table ID for nominal stress-strain data as a function of strain rate.*** | |
| 850 | 5.376E3 | 0 | 409 (Fig. B3) | |
| *Strain rate evaluation flag* | | ***Tensile stress evaluation*** | |  |
| Principal strain rates for each principal direction | | input via load curves with the tensile response corresponds to negative values of stress and strain | |  |


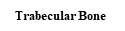
***
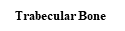

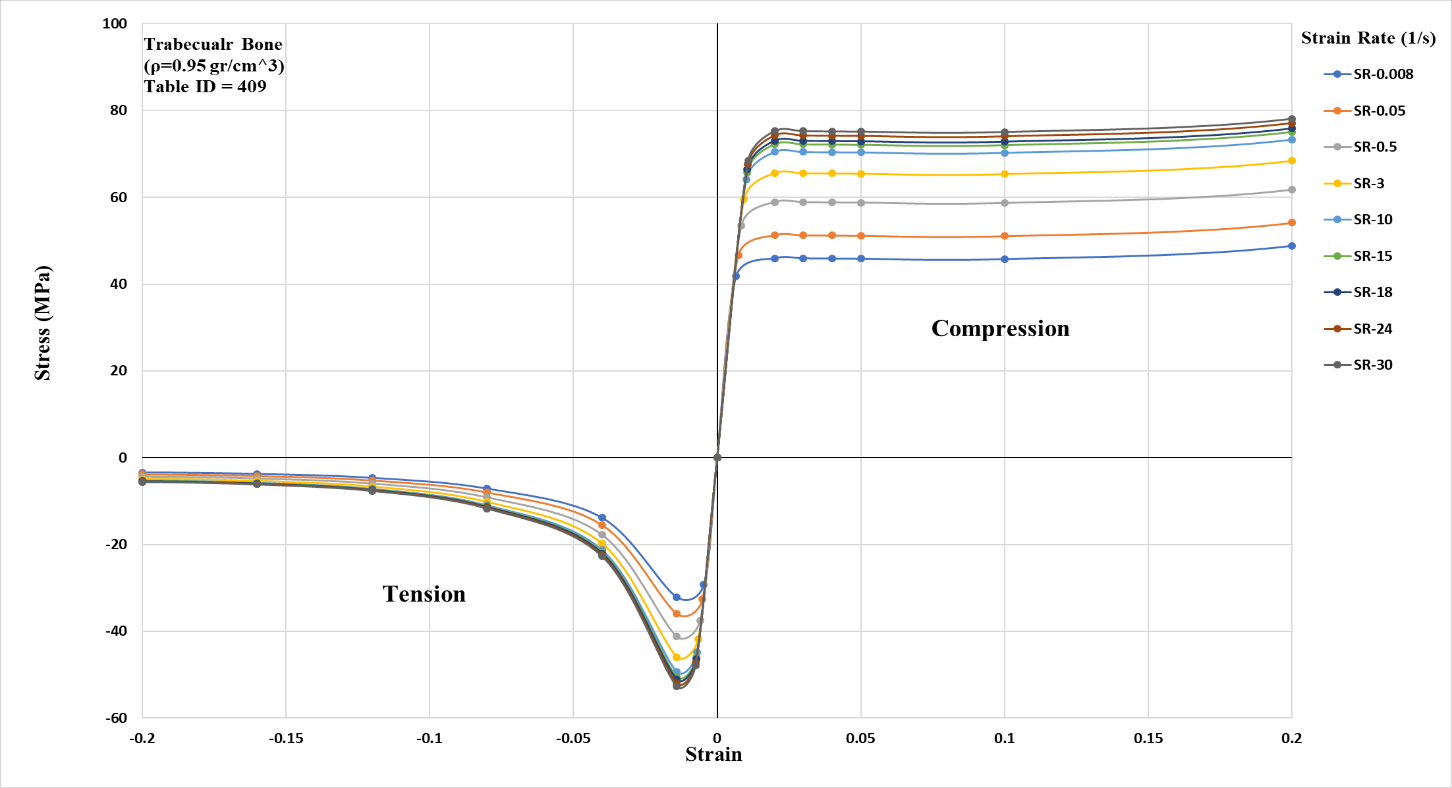
***
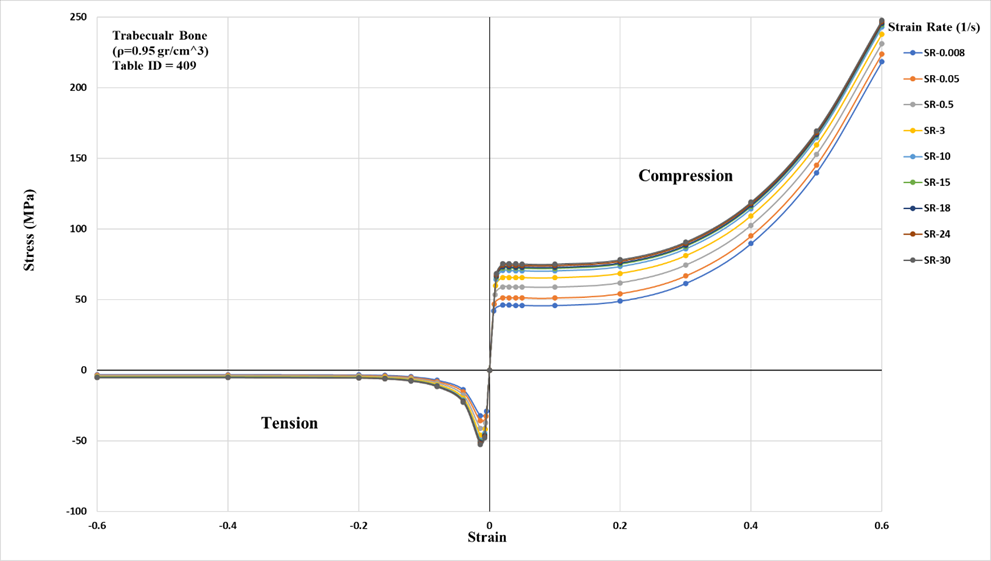


Figure A3. Nominal stress-strain curves for trabecular bone- Density =0.95 g/cm^3^ (Table ID=409)

| Trabecular Bone-T19 (ρ=1.79E-9) (MAT_FU_CHANG_FOAM (083)) | | | | |
| --- | --- | --- | --- | --- |
| *Density (kg/m^3)* | ***Young's modulus (MPa)*** | ***Rayleigh damping coefficient*** | ***Table ID for nominal stress-strain data as a function of strain rate.*** | |
| 1,790 | 1.648E4 | 0 | 419 (Fig. B4) | |
| *Strain rate evaluation flag* | | ***Tensile stress evaluation*** | |  |
| Principal strain rates for each principal direction | | input via load curves with the tensile response corresponds to negative values of stress and strain | |  |


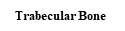
**
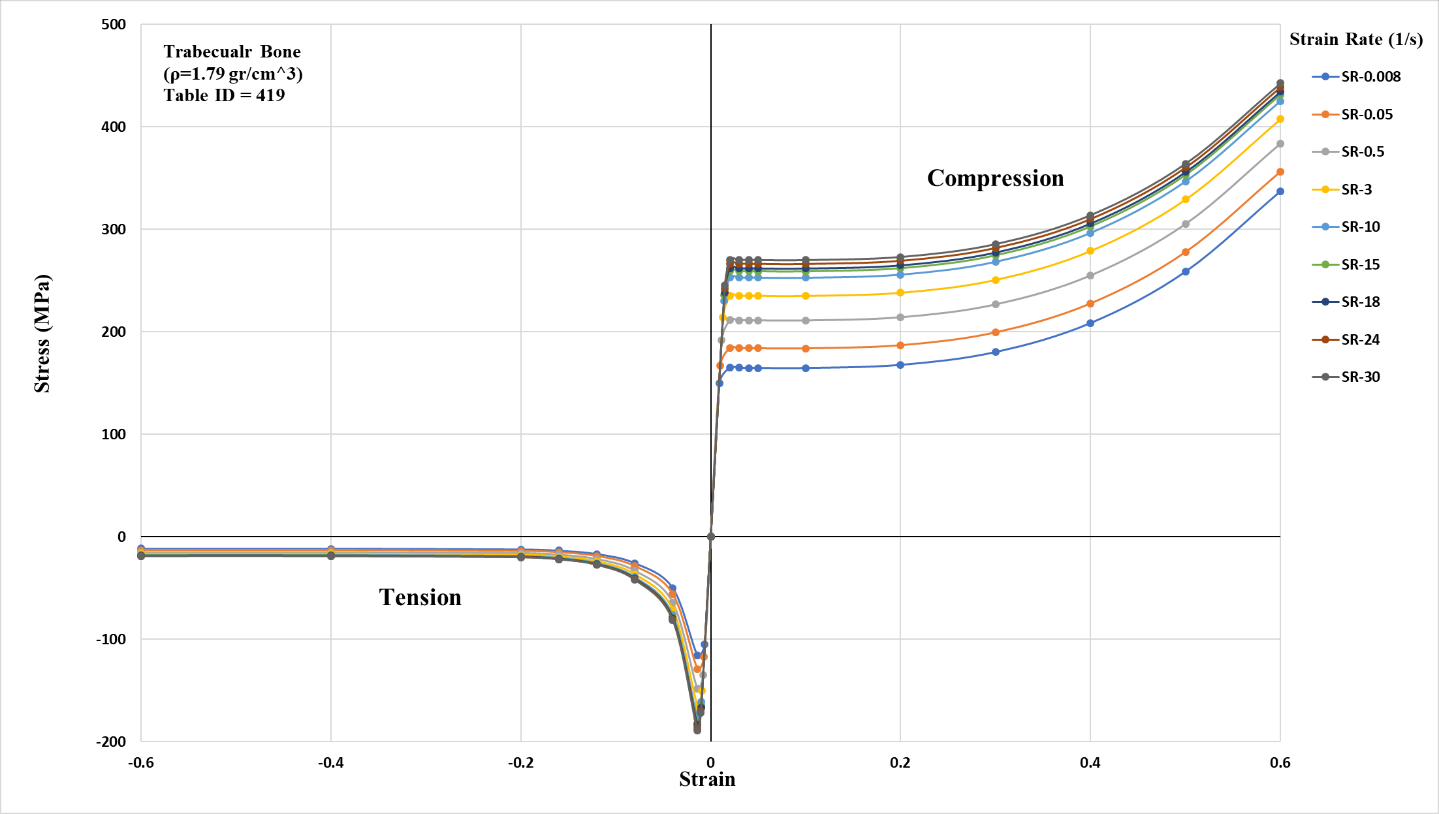
**


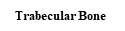
**
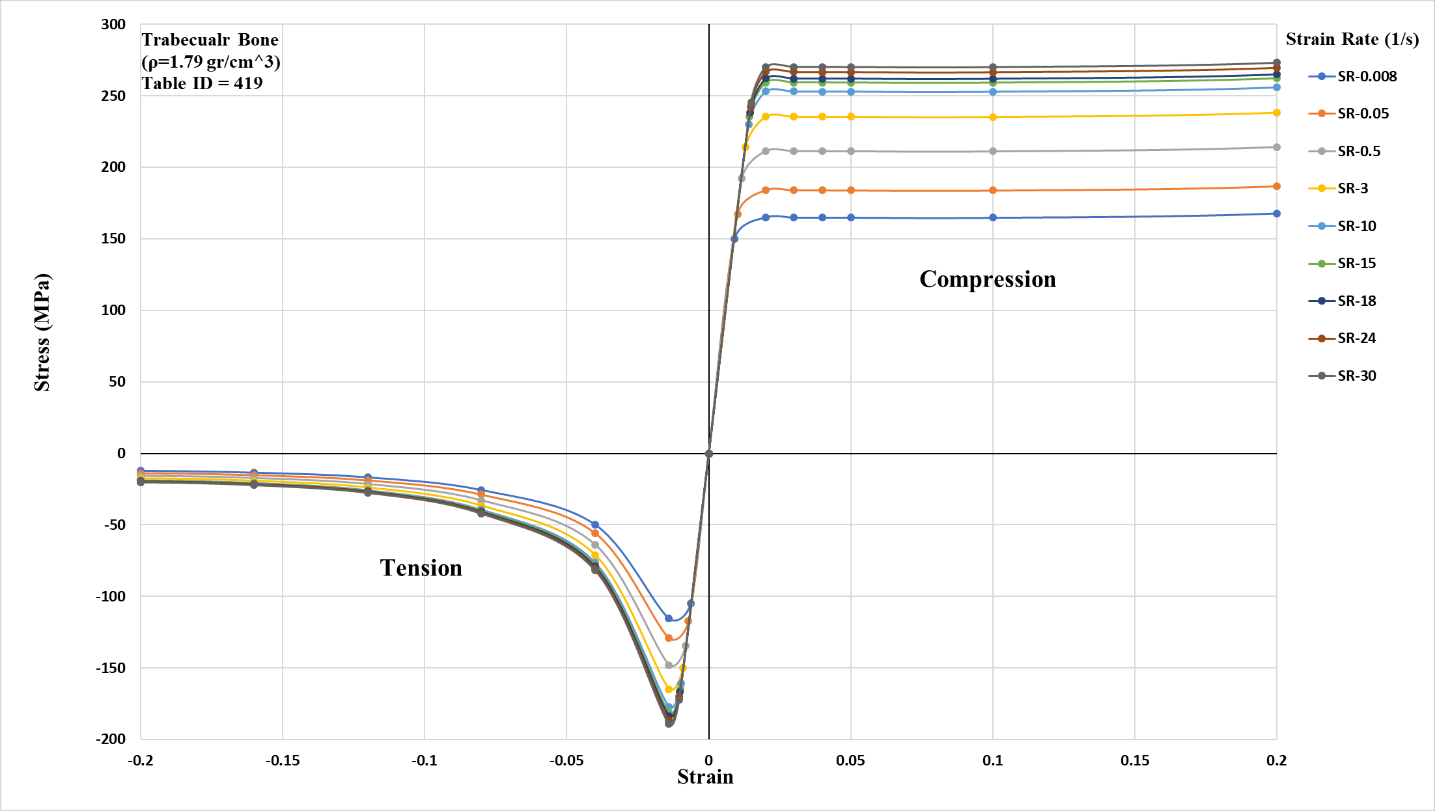
**

Figure A4. Nominal stress-strain curves for trabecular bone- Density =1.79 g/cm^3^ (Table ID=419)

| Trochanteric Soft Tissue (MAT_MOONEY-RIVLIN_RUBBER (027)) | | | | | | |
| --- | --- | --- | --- | --- | --- | --- |
| *Density (kg/m^3)* | ***Poisson's ratio*** | ***C10 (MPa)*** | ***C01 (MPa)*** |  |  |  |
| 920 | 0.4950000 | 0.085 | 0.02138 |  |  |  |

| Articular Cartilage (MAT_HYPERELASTIC_RUBBER (077_H)) | | | | | | |
| --- | --- | --- | --- | --- | --- | --- |
| *Density (kg/m^3)* | ***Poisson's ratio*** | ***C10 (MPa)*** | ***C01 (MPa)*** | ***C11 (MPa)*** |  |  |
| 795 | 0.4950000 | 0.352 | 0.306 | 0.052 |  |  |

| Sacroiliac Cartilage (MAT_MOONEY-RIVLIN_RUBBER (027)) | | | | | | |
| --- | --- | --- | --- | --- | --- | --- |
| *Density (kg/m^3)* | ***Poisson's ratio*** | ***C10 (MPa)*** | ***C01 (MPa)*** |  |  |  |
| 795 | 0.4950000 | 0.05 | 0.2 |  |  |  |

| Interpubic Disc (MAT_HYPERELASTIC_RUBBER (077_H)) | | | | | | |
| --- | --- | --- | --- | --- | --- | --- |
| *Density (kg/m^3)* | ***Poisson's ratio*** | ***Number of Prony series term*** | ***Shear modulus for frequency-independent damping (MPa)*** | ***C10 (MPa)*** | ***C01 (MPa)*** | ***C11 (MPa)*** |
| 1,200 | 0.4950000 | 2 | 0.5000000 | 0.05 | 0.2 | 0.25 |
| *shear relaxation modulus (MPa)* | ***decay constant*** | ***Shear modulus for frequency-independent damping (MPa)*** | | ***Limit stress for frequency independent, frictional, damping*** | | |
| 0.016 | 0.54 | 0.07 | | 0.06 | | |

***
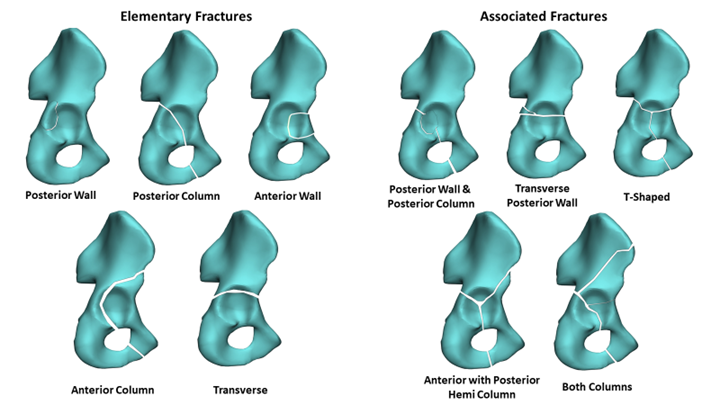
***

Figure A5. The Judet and Letournel acetabular fracture classification

***Appendix B. Transmitted load in the hip, sacroiliac, and pubic symphysis***

***
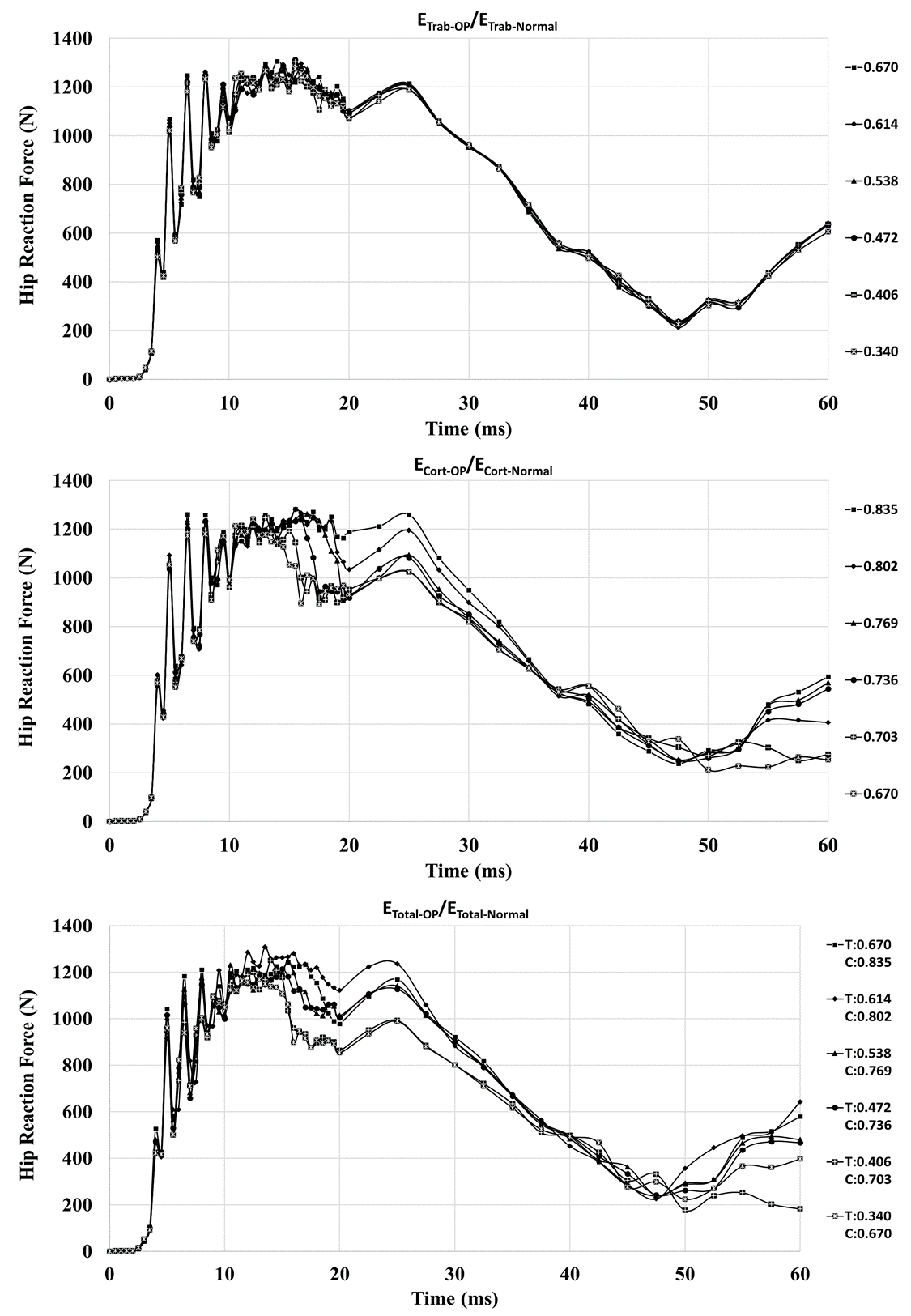
***

Figure B1. Effect of reduction in trabecular (E_Trab_), cortical (E_Cort_), and total (E_Total_) elastic modulus on the transmitted load within the hip joint
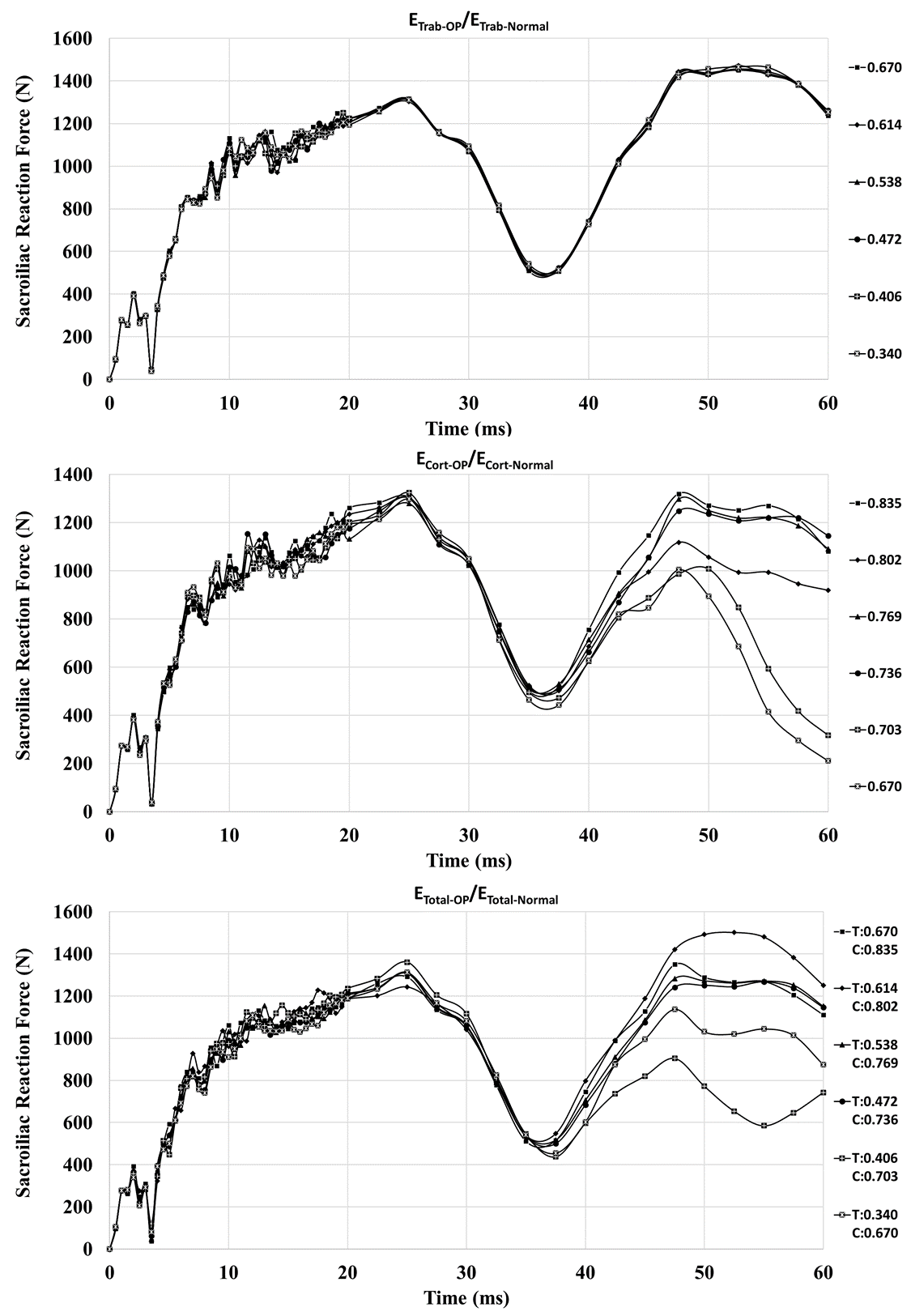


Figure B2. Effect of reduction in trabecular (E_Trab_), cortical (E_Cort_), and total (E_Total_) elastic modulus on the transmitted load within the sacroiliac joint.
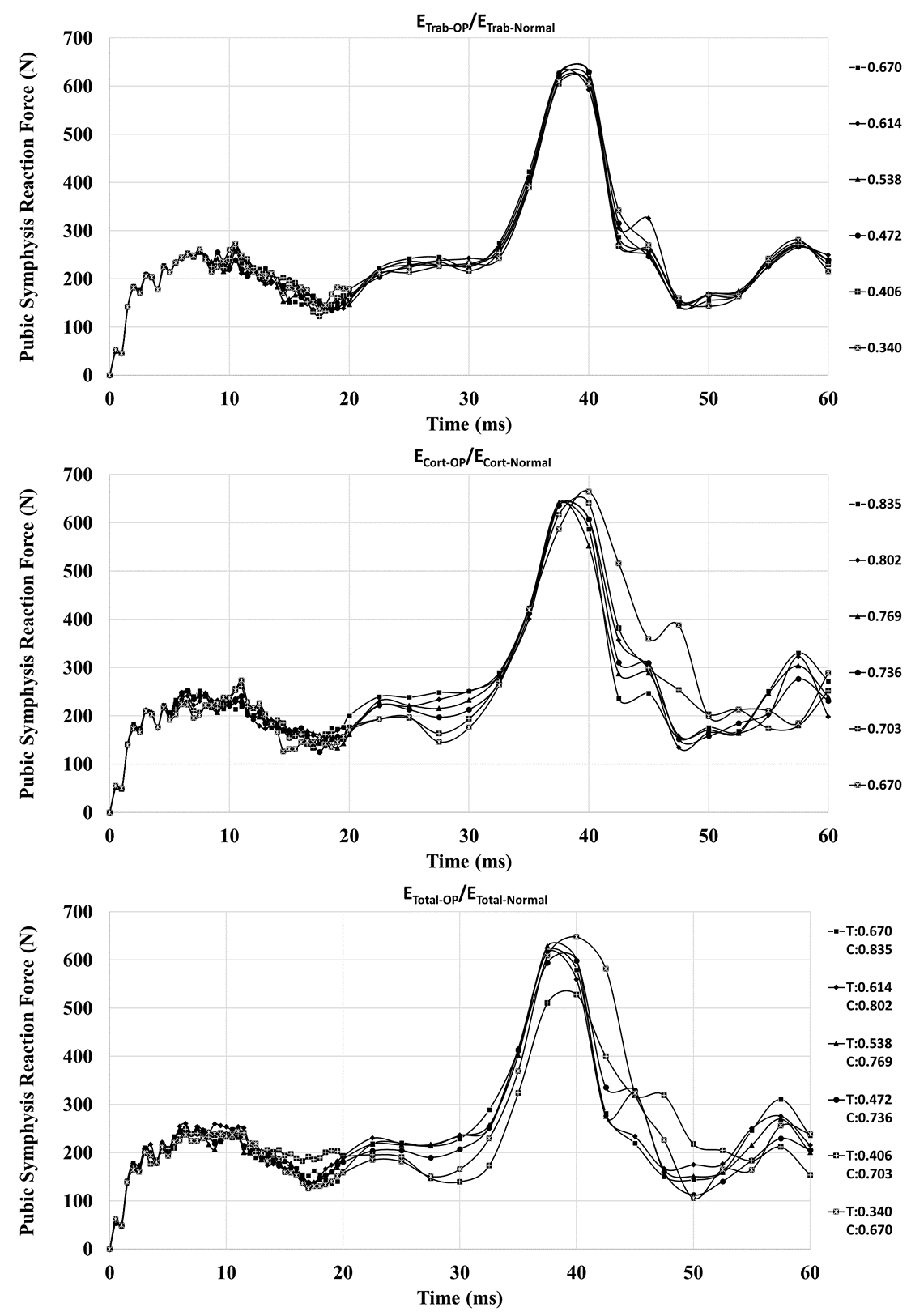


Figure B3. Effect of reduction in trabecular (E_Trab_), cortical (E_Cort_), and total (E_Total_) elastic modulus on the transmitted load within the pubic symphysis

***Appendix C (Model Validation)***


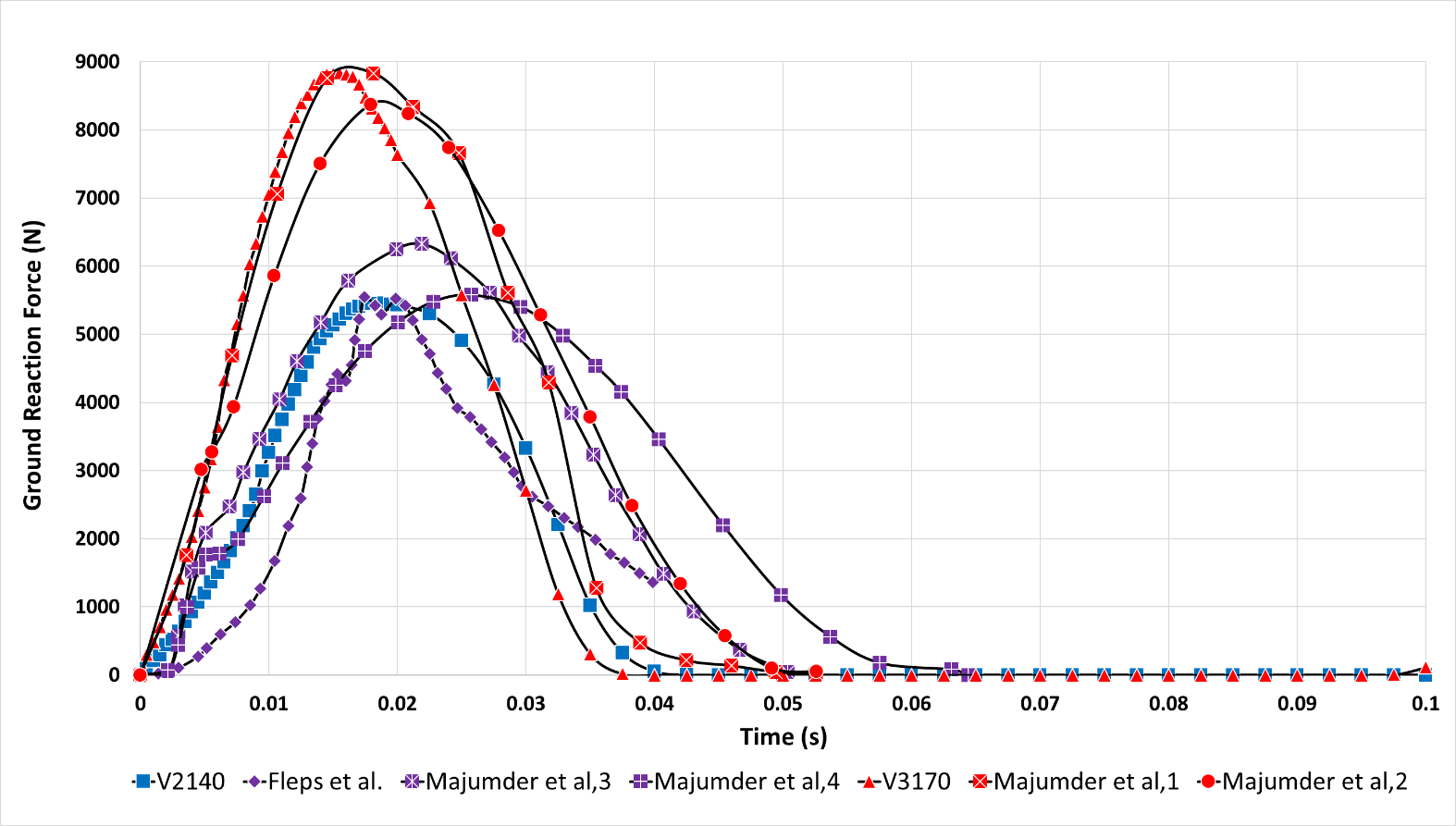


Figure C1. GRF: Current study (V_impact_ = 2140 and 3170 mm/s) vs. Fleps et al. [2] and Majumder et al.[3,4] for sideways fall experiments

Table C1. GRF_max_: Current study vs. previous studies

| **Researchers** | **V_Impact_ (mm/s)** | **GRF_max_ (N)** |
| --- | --- | --- |
| Current Study | 2140 | 5455 |
| Robinovitch et al. [5] | 1160-2580 | 1700-5600 |
| Robinovitch et al. [6] | 1400-6070 | 5600-8600 |
| Majumder et al. [7] | 1200-4790 | 3419-11540 |
| Current Study | 3170 | 8835 |
| Robinovitch et al. [6] | 1400-6070 | 5600-8600 |
| Majumder et al. [7] | 1200-4790 | 3419-11540 |
| Kroonenberg et al. [8] | 3350-4340 | 3720-9990 |

Reference

1. Enns-Bray WS, Bahaloo H, Fleps I, Ariza O, Gilchrist S, Widmer R, et al. Material mapping strategy to improve the predicted response of the proximal femur to a sideways fall impact. J Mech Behav Biomed Mater [Internet]. 2018;78(August 2016):196–205. Available from: https://doi.org/10.1016/j.jmbbm.2017.10.033

2. Fleps I, Enns-bray WSS, Guy P, Ferguson SJJ, Cripton PA, Helgason B, et al. On the internal reaction forces, energy absorption, and fracture in the hip during simulated sideways fall impact. PLoS One. 2018;13(8):1–18.

3. Majumder S, Roychowdhury A, Pal S. Simulation of hip fracture in sideways fall using a 3D finite element model of pelvis-femur-soft tissue complex with simplified representation of whole body. Med Eng Phys [Internet]. 2007 Dec 1 [cited 2018 Nov 8];29(10):1167–78. Available from: https://www.sciencedirect.com/science/article/pii/S1350453306002323

4. Majumder S, Roychowdhury A, Pal S. Hip fracture and anthropometric variations: Dominance among trochanteric soft tissue thickness, body height and body weight during sideways fall. Clin Biomech [Internet]. 2013;28(9–10):1034–40. Available from: http://dx.doi.org/10.1016/j.clinbiomech.2013.09.008

5. Robinovitch SN, Hayes WC, McMahon TA. Predicting the Impact Response of a Nonlinear Single-Degree-of-Freedom Shock-Absorbing System From the Measured Step Response. J Biomech Eng [Internet]. 1997 Aug 1;119(3):221–7. Available from: https://doi.org/10.1115/1.2796083

6. Robinovitch SN, Hayes WC, McMahon TA. Prediction of Femoral Impact Forces in Falls on the Hip. J Biomech Eng [Internet]. 1991 Nov 1;113(4):366–74. Available from: https://doi.org/10.1115/1.2895414

7. Majumder S, Roychowdhury A, Pal S. Effects of trochanteric soft tissue thickness and hip impact velocity on hip fracture in sideways fall through 3D finite element simulations. J Biomech [Internet]. 2008 Sep 18 [cited 2019 Oct 9];41(13):2834–42. Available from: https://www.sciencedirect.com/science/article/pii/S0021929008003424

8. van den Kroonenberg AJ, Hayes WC, McMahon TA. Dynamic Models for Sideways Falls From Standing Height. J Biomech Eng [Internet]. 1995 Aug 1;117(3):309–18. Available from: https://doi.org/10.1115/1.2794186
