# Supplementary material for: Effect of osteoporosis-related reduction in the mechanical properties of bone on the acetabular fracture during a sideways fall: A parametric finite element approach
Source: PLoS One. 2022 Feb 7;17(2):e0263458. doi: 10.1371/journal.pone.0263458 (PMC8820641; doi:10.1371/journal.pone.0263458)
Supplement: Attachment — Submitted filename: PONE-D-21-33350-Response to Reviewers.docx [file pone.0263458.s002.docx]

Dear Professor Tomaszewska,

Thank you for the opportunity to revise our manuscript entitled “*Effect of osteoporosis-related reduction in the mechanical properties of bone on the acetabular fracture during a sideways fall: A parametric finite element approach*”. We appreciate the careful review and constructive suggestions. The manuscript has certainly benefited from the insightful revision suggestions. Based on the comments from the referees, we have made changes to the manuscript, which are detailed below. We are confident that we have addressed all the comments carefully and provided clear responses.

Following this letter are the reviewers’ comments (in **bold**) with dedicated responses for each comment, including how and where the text is modified. The changes made to the manuscript are reported in *italics* in the response letter and marked with the Track Changes feature of the Microsoft office in the revised manuscript.

Your time and considerations are greatly appreciated.

Yours Sincerely,

Shahab Khakpour, on behalf of all the co-authors

Research Unit of Medical Imaging, Physics, and Technology (MIPT)

Faculty of Medicine, University of Oulu, Oulu, Finland

| **Reviewer #2:** | |
| --- | --- |
| **General Comment**  **The text is well written, clear and easy to comprehend and follow despite its length. I am enthusiastic about works like this and strongly recommend the publication of this work. Just some minor comments.**  **Response:**  We do appreciate your constructive and positive comments. We made our best to completely address your comments. | |
| **1** | **Consider unifying the nomenclature, e.g.:**  **- lowercase epsilon (table) or lunatic epsilon (Enns-Bray formulas) in App A.**  **- avoid to mix different methods of presenting numerical values**  **– L227-230 SI prefixes (ms) or decimal notation(10^(-8))**  **- E_Total-OP/E_Normal or E_Total-OP/E_Total_Normal throughout the main text and App A.**  **Response:**  Thank you for the comment. We unified the nomenclature and notations throughout the manuscript.  **Changes:**   - All epsilons in Appendix A were unified to the lowercase epsilon. - “s” was changed to “ms” in line 230 as follows:   “*3.36×10^-5^ ms* in the simulations”  Also, the units of horizontal axes in Fig B 1-3 were changed from s to ms.   - All E_Total-OP/E_Normal were changed to E_Total-OP/E_Total_Normal throughout the text, Fig 6-8, and Fig B 1-3 (appendix B). |
| **2** | **L218 Correct to “Green-St. Venant strain tensor”**  **Response:**  Thank you for the comment.  **Changes:**  The typo was corrected accordingly in Line 219 as follows:  “element softening (first principal strains of the *Green-St.Vernant strain* tensor higher than 0.014” |
| **3** | **Figs B1-B3 in App - Some decimal points in figures of total elastic modulus legend (on the right) are missing**  **Response:**  Thank you for the comment and for mentioning the needed change.  **Changes:**  The legends of Fig B1-B3 were crosschecked and corrected accordingly. |
| **4** | **References are not formatted in accordance with journal requirements**  **Response:**  Thank you for the comment.  **Changes:**  References were formatted according to the journal requirements (Vancouver style). |
| **5** | **Ref [15] – Journal title is missing**  **Response:**  We appreciate your comments.  **Changes:**  The reference was corrected. |
| **6** | **Figures 2-7 What do the circles mean ? Some explanation in figure’s footnote will be helpful.**  **Response:**  Thank you for the comment. Adding an explanation in the figure’s footnote would enhance the readability of the figures.  **Changes:**  Explaining statements were added to Figure 2-7 as follows:  “*Dashed circles: anatomical landmarks with a high risk of bone failure*” . |
| **7** | **Fig 3 and 6 – As no points can be seen on the most of the figures, also explanation in figure’s footnote will be helpful.**  **Response:**  Thank you for the comment. As mentioned by you, adding the figure’s footnote would help the reader to understand them better. We also think that you addressed Fig 5, instead of Fig 6.  **Changes:**  Additional explanation was added to Fig 3 and 5 as follows:  Fig 3: “*Neither tensile nor compressive cortical bone failure was observed.*”  Fig 5: “*The tensile and compressive strain concentrations were only minor.”* |
